# The environmental health citizen interview tool: towards an inclusive qualitative environmental wellbeing approach in support of planetary health

**DOI:** 10.3389/fpubh.2024.1462561

**Published:** 2024-11-21

**Authors:** Ben Delbaere, Evelise Pereira Barboza, Bianka Plüschke-Altof, Mariia Chebotareva, Evelien Van Rafelghem, Lauren Blockmans, Hans Keune

**Affiliations:** ^1^Department of Family Medicine and Population Health, University of Antwerp, Antwerp, Belgium; ^2^Instituto Salud Global Barcelona (ISGlobal), Barcelona, Spain; ^3^School of Economics and Business Administration, University of Tartu, Tartu, Estonia; ^4^School of Natural Sciences and Health, Tallinn University, Tallinn, Estonia; ^5^IDEWE, Leuven, Belgium

**Keywords:** planetary health, environmental wellbeing, epistemic justice, qualitative assesment, diversity and inclusion, urban green and blue infrastructure, nature based solutions

## Abstract

**Background:**

Nature-based Solutions (NbS) are vital for addressing climate change and biodiversity loss, yet their benefits are often unequally distributed. This study introduces the Environmental Health Citizen Interview Tool, aiming to inclusively capture diverse perspectives on environmental wellbeing using qualitative research methods. The principles of epistemic justice and planetary health are central to its development.

**Methods:**

The Environmental Health Citizen Interview Tool was developed as a qualitative tool, incorporating a simple visualization scoring system for responses. Six environmental health determinants were selected, with in-depth open-ended follow-up questions. Content validation involved transdisciplinary expert consultation. A guidebook for using the tool was also developed.

**Findings:**

The tool offers a comprehensive approach to inquire environmental wellbeing, accommodating diverse perspectives through in-depth inquiries. Limitations include the need for further validation and testing.

**Interpretation:**

The Environmental Health Citizen Interview Tool provides a practical framework for inclusive assessment of environmental wellbeing, aligned with planetary health and epistemic justice principles. Its application should be complemented by quantitative environmental monitoring such as air quality and be contextualized by local researchers for reliability and relevance. Future research should focus on refining the tool and exploring its utility in diverse settings to inform equitable local policy interventions.

## Introduction

Nature-based Solutions (NbS) play an important role in mitigating pressing societal challenges such as climate change adaptation, equity, environmental change and, biodiversity loss (1). Moreover, they can improve environmental conditions in both urban and rural areas (2). These improvements are however not evenly distributed among the population (3, 4). Therefore, designers and implementers of NbS must consider issues such as by, for and with whom the NbS are implemented and who benefits from the NbS (5, 6). To operationalize these questions means to develop pathways and instruments that enables an understanding of how local communities experience their environment, which environmental conditions burden and/or support them and which changes they detect. There is thus a need to inquire how diverse populations experience their wellbeing in relation to the environment. Developing culturally sensitive tools that account for epistemic justice could be such a pathway (7).

Epistemic justice is rooted in the recognition that knowledge is often produced, validated and disseminated within social structures that perpetuate injustices such as systematic biases, discrimination and exclusion, and therefore seeks to ensure that diverse perspectives and forms of knowledge are acknowledged, valued and incorporated in to the broader academic discourse (8). Hence, qualitative research is useful to develop tools for capturing diverse conceptualizations of environmental wellbeing by allowing participants to elaborate on their perspectives without the researcher imposing conceptualizations upon them.

### Environmental conditions in cities and the role of the NbS

Environmental conditions such as air pollution, excess heat, noise pollution, and access to urban green and blue spaces play pivotal roles in shaping public health outcomes. Air pollution, for instance, has been linked to a staggering number of preventable deaths annually, emphasizing the critical need for adherence to WHO guidelines (9). Similarly, excess heat, often exacerbated in urban areas, poses significant health risks, leading to premature deaths and highlighting the importance of understanding public perception to inform effective policy interventions (10, 11). Noise pollution, another pervasive environmental hazard, has far-reaching health implications, including sleep disturbances and cardiovascular diseases, underscoring the necessity for compliance with established guidelines (12). Moreover, the availability and quality of urban green and blue spaces have been shown to profoundly impact both mental and physical well-being, with inadequate access contributing to preventable deaths (4). Addressing these environmental determinants comprehensively is crucial for promoting public health and fostering sustainable urban environments. NbS offer promising avenues for mitigating the adverse effects of environmental determinants such as air pollution, excess heat, noise pollution, and inadequate access to green spaces in urban areas (2). NbS involves utilizing and enhancing natural features and ecosystems to address environmental challenges while providing additional benefits for communities. For instance, strategic planting of trees and vegetation can help reduce excess heat by providing shade, cooling urban microclimates, and mitigating the urban heat island effect (1). Noise pollution can be mitigated through the strategic placement of green buffers and sound-absorbing vegetation, which can act as natural barriers to absorb and diffuse anthropogenic noise. Furthermore, increasing access to urban green and blue spaces through NbS initiatives like creating parks, community gardens, and restoring water bodies not only enhances physical and mental well-being but also contributes to biodiversity conservation and ecosystem resilience (13).

### Conceptualizing environmental wellbeing

Firstly, when conceptualizing wellbeing, a duality can be observed between what is called ‘objective’ and ‘subjective’ wellbeing. ‘Objective’ wellbeing refers to “a comparison of life circumstances with social norms and values” (14), thus on a societal level. Whereas ‘subjective’ wellbeing includes “psychological functioning and affective states” (14) which are linked to the individual. When adressing ‘subjective wellbeing’, three conceptualizations are prominent: (1) hedonic wellbeing, which refers to everyday emotions such as anger, sadness and happiness, (2) eudemonic wellbeing, which deals with the purpose and meaning of a person’s life, and (3) life evaluation, which focusses on the quality or goodness of life (15, 16).

We understand the concept of ‘environmental wellbeing’ as an elaboration of subjective wellbeing that encompasses factors such as hedonic wellbeing and life evaluation. Our conceptualization aknowledges that the understanding of wellbeing is different across different cultures, geographical areas and specific subgroups of the population and is therefore somewhat fluid. Hence, we also understand environment as the spatial suroundings including natural/non-natural landscapes and cultural relationships.

In understanding the complex interplay between individuals and their environments, the role of institutional and social determinants emerges as important factors influencing overall well-being. When considering institutional determinants in relation to the environment, it becomes imperative to explore the extent to which a diverse range of residents and public sector actors are involved in the decision-making processes that shape urban landscapes. Moreover, social determinants underscore the role of social cohesion, engagement, and identity within communities. As loneliness has been linked to various adverse health outcomes, understanding and fostering social interactions at the neighborhood level became paramount in promoting environmental wellbeing.

We draw from the extensive work of Henrich, Heine (17) to contest that constructs related to human psychology and behavior, such as wellbeing can make any claims to universality. That is to say, evidence on nature and wellbeing is mainly informed by research from Western, Educated, Industrialized, Rich and Democratic (WEIRD) societies (7) resulting in an underrepresentation of human diversity. This underrepresentation of human diversity is in essence a planetary health problem (7). This is because planetary health is intertwined and contingent on the ethnosphere. Moreover, planetary health underlines the importance of human rights and equality (18). Hence, inquiring environmental wellbeing for planetary health needs further understanding about how different people experience diverse environmental conditions. This tool was created in the context of the Horizon2020 GoGreenRoutes project, which aims to introduce NbS through participatory decision-making in six cities from different backgrounds. Hence, despite being focused on the European context, in this study we propose a tool that could represent different populational backgrounds within Europe to include post-socialist societies, such as the baltic states, and the Balcanic Peninsula. Given the need to create culturally sensitive instruments and tools that account for epistemic justice (7), a particular interest is placed in minority populations that are often not reflected in mainstream academic outputs on the environmental and wellbeing fields.

## Tool development

The research team consisted of interdisciplinary experts in environmental health, public health, psychology, nursing, urban sociology and economy. The tool was developed based on consensus meetings within the research team. The final version consists of 12 closed-ended questions to be scored on a scale from 1 to 6, 11 open-ended follow-up questions adressing reciprocity and, 11 socio-demographic and diversity questions.

### Operationalizing environmental wellbeing

Although many subjective wellbeing measures exist and are being used to measure wellbeing in relation to green spaces and the environment (19), a lack of pluralism in conceptualizations and operationalizations of wellbeing prevails (7). In relation to ‘environmental wellbeing’, six environmental determinants were selected during consensus meetings with the research team: (1) air quality, (2) excess heat, (3) excess noise, (4) urban green and blue spaces, (5) institutional determinants, (6) social determinants (Table 1). Therefore two questions regarding each of the components were included in the tool. The first question was focused on the perception of quality of each environmental condition, or the level of political engagement and social interaction. The second question was focused on the perception in terms of the effect of each dimension on wellbeing. Inspiration was drawn from the concept of positive health (20) to create a tool that allows numerical scoring in order to create a conversational focus for participants to elaborate on their understanding of the environmental determinants that related to their wellbeing. The scoring then appears on a ‘spiderweb’ graph which visualizes and mirrors the answers of participants and which can be used in conversation to refer to. This scoring is merely a prompt to focus the interview, rather than to be quantitatively analyzed.

**Table 1:** Operationalization of environmental wellbeing through the scoring of environmental wellbeing determinants.

Individual environmental health determinant assessment					
Air pollution					
1) How would you score the air pollution in your neighborhood?					
low	relatively low	rather low	rather high	relatively high	High
1	2	3	4	5	6
2) To what extent does the air pollution in your neighborhood affect your wellbeing?					
1	2	3	4	5	6
very little	little	rather little	rather partially	partially	almost fully
Excess heat					
3) How would you score the level of excess heat in your neighborhood?					
1	2	3	4	5	6
low	relatively low	rather low	rather high	relatively high	High
4) To what extent does excess heat in your neighborhood affect your wellbeing?					
1	2	3	4	5	6
very little	little	rather little	rather partially	partially	almost fully
Excess noise					
5) How would you score the level of excess noise in your neighborhood?					
1	2	3	4	5	6
low	relatively low	rather low	rather high	relatively high	High
6) To what extent does excess noise in your neighborhood affect your wellbeing?					
1	2	3	4	5	6
very little	little	rather little	rather partially	partially	almost fully
Urban green and blue spaces					
7) How would you score the quality of the green and blue spaces in your neighborhood?					
1	2	3	4	5	6
very poor	poor	rather poor	rather good	good	very good
8) To what extent do the green and blue spaces in your neighborhood affect your wellbeing?					
1	2	3	4	5	6
very little	little	rather little	rather partially	partially	almost fully
Institutional determinants					
9) To what extent are you involved in activities that enhance the living/environmental quality in your neighborhood?					
1	2	3	4	5	6
not at all involved	not involved	rather not	rather involved	involved	very involved
10) To what extent does your involvement in activities that enhance the living/environmental quality of your neighborhood affect your wellbeing?					
1	2	3	4	5	6
very little	little	rather little	rather partially	partially	almost fully
Social determinants					
11) Do you interact with people from your neighborhood (besides relatives)?					
1	2	3	4	5	6
almost never	rarely	rather rarely	rather often	Often	very often
12) Does the interaction with people from your neighborhood affect your wellbeing?					
1	2	3	4	5	6
very little	little	rather little	rather partially	partially	almost fully

Cities are hotspots of noise, heat island effects, lack of green space and of air pollution (21), each of these environmental determinants was therefore included in the operationalization of environmental wellbeing.

1. Air pollution is a significant environmental determinant of health. Compliance with the WHO air pollution guidelines for PM2,5 and NO2 was estimated to prevent, respectively, over 100,000 and 50,000 deaths annually (9, 22). However it remains unclear how air quality is perceived to impact wellbeing.
2. Excess heat in cities is a well-known issue that is often referred to as the urban heat island (UHI). A recent health impact assessment estimated that the UHI in 93 European cities contributed to 1,5°C temperature increase during the summer of 2015 and 6,700 premature deaths were attributable to this effect during summer months (11). Even though the adverse health outcomes of excess heat are well documented (23) it remains largely unclear how excess heat is perceived by urban dwellers – e.g. do they perceive excess heat as a health hazard? This information can inform policy makers to establish targeted risk awareness strategies (10).
3. Excess noise has been shown to be an environmental hazard to health that impacts, among other things, sleep disturbance, cardiovascular and metabolic disease and adverse birth outcomes (24–26). It was estimated that almost 60 million adults are exposed to harmful (road traffic) noise levels in European cities (over 40% of the adult population). Compliance to WHO guidelines could prevent around 3,600 deaths from ischeamic heart disease anually (12).
4. Urban green and blue spaces impact human health in divers and complex ways (27). Urban green and blue spaces have shown positive effects on both mental and physical human health (13, 28). However, it was estimated that over 60% of the population has insufficient access to green spaces in European cities (4). Hence, achieving WHO recommendation for universal access to green spaces could prevent up to 43,000 deaths each year (4). It’s important to mention that evidence on mental health and wellbeing is largely dependent on research conducted in WEIRD countries (7). As multidimensional quality conditions of urban green and blue spaces influcence time spent and type of use by different subgroups of the population (29). This enables the identification of diverse multidimensional quality conditions that reflect human diversity beyond the WEIRD conceptualization.
5. ‘Institutional determinants’ such as political engagement toward the direct living environment is an important component for autonomy and sense of belonging. Building on the Gender, Inclusion and Diversity framework for Nature-based Solutions in cities (5), it is important to understand to what extend a diverse set of residents, as well as different actors from the public sector, are involved in the political process of shaping the physical environment (5). Consequently, questions inquiring people’s involvement and the impact this involvement has on their wellbeing were included.
6. Lastly, ‘neighborhood social interaction’ reflects components of social cohesion, engagement and identity. As loneliness has been associated with adverse effects on all health outcomes (30), and can be remediated – in part – at the community level (31), questions on social interaction at the neighborhood level were included. Additionally, a question was added to understand how this social interaction at the neighborhood level affects people’s wellbeing.

### In dept follow up questions addressing reciprocity

Applying a planetary health perspective, it is imperative to address environmental wellbeing in a reciprocal way, ie, beyond a onedirectional environment-to-human perspective. We therefore included questions assessing people’s perceived impact on the environment. Thus, in depth follow up questions were developed in four distinct categories: (1) sustainable behavior, (2) climate change, (3) quality aspects of green and blue spaces, and (4) critical consciousness.

1. We introduce ‘sustainable behavior’ as a broad umbrella term that captures planetary health solutions at the individual level, eg, taking care of the natural environment, using active forms of transportation, visiting green and blue spaces as leisure activity. That is to say, behavior that has a positive impact on human health and stays within safe planetary bounderies or helps restore planetary boundaries that have already been transgressed (e.g., restoring biodiversity, closing nutrient cycles). Open questions on the above topics allows participants to elaborate on what they believe to be ‘sustainable behavior’ without imposing a conceptualization on them.
2. Perceptions around climate change are usually measured in three distinct categories: believes about the reality of climate change, causes of climate change, and consequences of climate change (32). This tool focuses on the latter by asking people how concerned they are about the consequences of climate change on their own city. The open-ended nature of this question allows for elaboration on what people perceive to be consequences of global climatic change at the local city level.
3. Quality aspects of green and blue spaces are also included to identify and understand the multidimensional quality criteria relevant to the context, the tool includes questions on what people think about the green and blue spaces in their neighborhood, if and how they use these spaces and why. In additon, questions regarding the perceptions of the non-natural environment (e.g., buildings, streets) and the difference in behavior as a result of different environments (e.g., natural versus non-natural) are included. Moreover, this tool inquires how people perceive that the quality of urban green and blue spaces has evolved over time to address possible changes due to temporal aspects (33).
4. As this tool was created with the intention to acount for epistemic justice, assessing critical consciousness in relation to environmental wellbeing is crucial. The construct of critical consciousness was first introduced by Freire (34) in the understanding of awareness about ones (marginalized) position in a certain society and the forces of power that establish and maintain these structural inequalities. In practice, this tool inquires whether people believe that their health and wellbeing is influenced by their social position and how empowered they feel to tackle environmental issues.

### Addressing human diversity through socio-demographic questions

In order to understand how a diverse set of residents experiences and conceptualizes environmental wellbeing, it is pivotal to explicitly assess the level of diversity within the sample beyond age and gender. We acknowledge that inquiring about rather private issues such as self-identified gender identity or sexuality might be somewhat intrusive, however, in order to achieve epistemic justice, identifying minority populations is imperative. Eleven socio-demographic questions were included based on the gender, inclusion and diversity framework for nature-based solutions in cities – see Table 2.

**Table 2:** Addressing human diversity through socio-demographic questions.

Demographic variables	
How old are you?	
What is your self-identified national identity?	1. Irish
	2. Finnish
	3. French
	4. Bulgarian
	5. Estonian
	6. Swedish
	7. Ukrainian
	8. Russian
	9. Other
What is your self-identified gender identity?	1. Non-binary
	2. Gender fluid
	3.Female
	4. Male
	5. Other
	6. Prefer not to say
Do you self-identify with one or several of the following characteristics?	1. Ethnic minority
	2. living with disability
	3. Identifying as LGBTQI+
	4. Combination of 1, 2 and 3
	5. Combination of 1 and 2
	6. Combination of 1 and 3
	7. Combination of 2 and 3
	8. None
In which country were you born?	1. Republic of Ireland
	2. Finland
	3. France
	4. Bulgaria
	5. Estonia
	6. Sweden
	7. Ukrainian
	8. Russian
	9. Other
	10. Prefer not to say
In which country were you born?	1. Republic of Ireland
	2. Finland
	3. France
	4. Bulgaria
	5. Estonia
	6. Sweden
	7. Ukrainian
	8. Russian
	9. Other
	10. Prefer not to say
In which country were you born?	1. Republic of Ireland
	2. Finland
	3. France
	4. Bulgaria
	5. Estonia
	6. Sweden
	7. Ukrainian
	8. Russian
	9. Other
	10. Prefer not to say
In which neighborhood do you live?	
What is your current employment status?	1. Employee
	2. Self-employed
	3. No paid work
IF no paid work	1. Unemployed
	2. Sickness or invalidity
	3. Studies
	4. Retirement
	5. I do the housekeeping, without benefits
	6. I am a family worker
	7. Other situation, describe
Highest level of educational attainmen	1. No diploma
	2. Lower education
	3. Secondary education
	4. Bachelor
	5. Master
	6. Doctorate with thesis
	7. Other diploma, specify

Self-identified national identity is inquired alongside the country of birth, as both do not necessarily overlap. The options provided need to be adapted to the context where the tool is employed. For the development of the tool we included common self identified national identities and countries of birth from the cities involved in the GoGreenRoutes project, wherein this tool was developed. As for minority status, it is asked if one self-identifies as ethnic minority, living with disability or identifies as LGBTQI+. This with an option for a combination of characteristics to account for intersectionality. Lastly, questions around employment status and highest level of educational attainment are included.

### Content validation through transdisciplinary expert consultation

To make sure that the tool measured what it intends to measure, the researchers sought to validate the content of the questions by a transdisciplinary pool of experts. Therefore, a pilot workshop was organized in Maynooth, Ireland (February 2023). Ten experts, both academic and societal, from different fields, nationalities and cultural backgrounds participated in the workshop. Academic participants had expertise in: environmental health, public health, psychology, nursing, urban sociology and economy. Societal participants worked for NGOs or were civil servants at the municipal level of medium sized European cities from six different countries and had expertise in: mental health, citizen engagement and participatory approaches. The workshop was structured in three consecutive parts, (1) introducing the tool and its conceptualization, (2) applying the tool through roleplay, and (3) providing feedback.

(1) To allow for a proper understanding of the tool and its conceptualization, the materials were delivered to the participants beforehand through email. This enabled the first part of the workshop to be interactive where participants could ask targeted questions for clarification after a brief introduction of the tool. (2) Next, participants were divided in to three groups to apply the tool during a roleplay. Participants took turns in playing different roles (interviewer, interviewee, and observer). The observer was asked to take notes about the practical application of the tool while interviewer and interviewee were asked to comment on the content and clarity of the questions. (3) Lastly, feedback was provided by participants on both content and practical application of the tool, both orally and written. Final adjustments were made based on their comments and suggestions.

### Developing a guidebook

A guidebook (Supplementary file 1) consisting of three core elements was developed to ensure proper use of the tool. The first core element describes the goal, content and methodological context of the tool. The second element entails key aspects to consider when conducting qualitative research such as, inductive process, importance of context, importance of the emic perspective – that is the viewpoint of the insider, and the importance of reflexivity (35). The last element consists of a fieldwork protocol that describes the use of the tool step by step. In addition, an online training course was developed and was delivered to civil servants at the municipal level for further application of the tool. This could potentially positively influence the design of participatory procesesses at the municipal level, and the understanding of the needs and wants of local populations at baseline before undertaking green space interventions.

## Usage of the environmental health citizen interview tool

After the validation through transdisciplinary expert consultation, the tool was finalized. It is proposed to apply the tool with individuals present in the area of interest, including adults visiting the area or living in close proximity. Depending on local requirements, the participation would be dependent on a written informed consent form. Convenience sampling takes place until saturation of information occurs. Interviews can be recorded if applicable and transcribed integrally and verbatim, to prevent data reduction. Moreover, data should be pseudo-anonymised (ie, names will not be retrieved). Also, researcher triangulation is strongly suggested during data collection and analysis. For the interview process, two applicants should be involved: interviewer one (I1), who is fluent in the local language, and interviewer two (I2), a researcher providing methodological and logistical support (eg, audio recording).

### Environmental wellbeing and awareness

**Figure 1:**
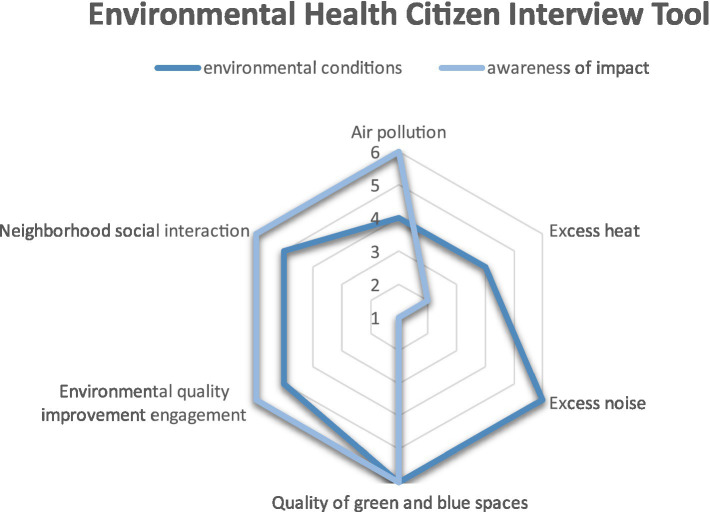
Example of the individual ‘spiderweb’ after scoring the questions. This figure can be used to guide and focus the conversation around environmental wellbeing during an individual interview, with 1 meaning ‘low’ and, 6 meaning ‘high’.

First, the participant is asked to score the questions as outlined in Table 1, which generates a ‘spiderweb’ as displayed in Figure 1. The spiderweb is shown to the participant, which facilitates the interview about the local environmental conditions and participants’ awareness about the impact of those conditions.

### In-depth follow up questions

After the diagloge faciliated by the spiderweb has concluded, the interviewers proceed with the open-ended segment of the semi-structured interview - see Table 3. Allowing for flexibility in questioning and, encouraging participants to elaborate freely. Additional questions for clarification are asked as needed.

**Table 3:** In depth follow up questions addressing reciprocity.

Additional open questions
Sustainable behavior
1. How do you engage in sustainable activities? e.g. actively taking care of the natural environment? 2. Do you ever walk or cycle? If yes, why do you walk/cycle (to commute, or for pleasure)? Do you usually use a route next to green/blue spaces? Why (faster, nicer, shorter…)? If not, why not? Security reasons, lack of facilities, mobility problems…? 3. What other green/blue spaces do you usually visit during the week/weekend (e.g. parks, forest, canals, lakes, beach, etc.)? Why? Could you describe it?
Climate change
4. How concerned are you about the impact of climate change on your city?
Quality aspects of green and blue spaces
5. What do you think about natural environments (green/blue spaces) in your neighborhood? And in particular, this area. 6. Do you use these spaces (and in particular this area)? Why? Why not? What activities do you do? 7. What do you think about the non-natural (artificial) environment in your neighborhood? (E.g. buildings, streets, services, traffic, etc.) 8. Has the natural environment in your neighborhood changed over time? How has it changed? Has it improved/gotten worse? 9. Do you think your behavior or well-being is related to the type of environment in which you are? How do you think it is related? Could you tell me an example?
Critical consciousness
10. How empowered do you feel to take action to tackle environmental issues in your neighborhood? 11. Do you believe that your social position within society influences your health and wellbeing? Why/Why not? How?

### Socio-demographic and diversity questions

Lastly, participants are asked to complete a form containing socio-demographic questions - see Table 2. The socio-demographic and diversity questions are based on the local specificities of our areas of interest given the GGR project. Further application in other settings should adjust the information to better fit the local context.

## Discussion

### Limitations

The present study and its associated tool development exhibit some limitations. Firstly, we did not address light pollution, despite its known repercussions on human health, wildlife, and plant life (36). Incorporating this aspect would enrich the concept of environmental wellbeing, particularly within the framework of Planetary Health. However, this aspect was not explicitly included because a selection of aspects had to be made not to overwhelm the participants with too many questions and other aspects were deemed more relevant.

Secondly, although we inquire about sustainable behavior, we recognize the imperative for transformative change at a systemic level to effectively address environmental challenges. Thirdly, it is acknowledged that the tool could enhance its robustness through formal content validation procedures, such as employing a Delphi method involving environmental health experts and societal stakeholders.

### Recommendations for future research and application of the tool

Ideally, the tool should be applied in combination with quantitative assesments of environmental conditions such as ambient temperature, soundscape, etc. When applying the tool, it should always be contextualized by local researchers and local societal actors that understand the cultural sensitivities. Moreover, the qualitative analysis of the interview data needs to be triangulated by having local researchers analyse the data independantly from other researchers in order to improve rigor and thrustwortiness of the interpretations. The authors will attempt to apply the tool in an upcoming study, in combination with above mentioned assessments of environmental conditions.

## Conclusion

The Environmental Health Citizen Interview Tool represents a novel approach to understanding environmental wellbeing through an inclusive qualitative lens, aligning with the principles of planetary health. By focusing on the subjective experiences of diverse populations, this tool addresses the limitations of traditional quantitative measures and recognizes the importance of epistemic justice in environmental research. A crucial aspect of the novelty of the tool is its attention to human diversity, as reflected in the socio-demographic questions included. By explicitly assessing characteristics such as ethnicity, disability, and LGBTQI+ identity, the tool aims to capture the perspectives of minority populations often marginalized in mainstream research. Furthermore, this contextualized qualitative tool can serve as an accessible pathway for local governments to explore and comprehend local needs regarding environmental wellbeing. This understanding can facilitate the development of targeted strategies aimed at enhancing local environmental conditions. In conclusion, the Environmental Health Citizen Interview Tool offers a promising approach to understanding environmental wellbeing from an inclusive and qualitative perspective. By centering the voices of diverse populations and addressing the complexities of human-environment interactions, this tool has the potential to inform policy, planning, and interventions aimed at promoting planetary health and social equity.

## Data Availability

The raw data supporting the conclusions of this article will be made available by the authors, without undue reservation.
