# Supplementary material for: The environmental health citizen interview tool: towards an inclusive qualitative environmental wellbeing approach in support of planetary health
Source: Front Public Health. 2024 Nov 21;12:1462561. doi: 10.3389/fpubh.2024.1462561 (PMC11618830; doi:10.3389/fpubh.2024.1462561)
Supplement: Supplementary file 1 [file Supplementary_file_1.docx]

**ENVIRONMENTAL HEALTH CITIZEN INTERVIEW TOOL GUIDEBOOK**

The ENVIRONMENTAL HEALTH CITIZEN INTERVIEW TOOL was developed to measure changes in individual health perceptions before and after nature-based solutions. The Environmental Health Citizen Interview Tool is an instrument to gather qualitative data. The tool consists of 11 demographic variables, 12 items to be scored on a 6 point Likert-scale divided over 6 topics: **air pollution, excess heat, excess** **noise, quality of green and blue spaces, environmental quality improvement** **engagement,** and **neighborhood social interaction.** Both perceptions on these topics as well as the impact these topics have on individual wellbeing will be mapped in a spider web diagram. This diagram is a means to start the conversation for qualitative data collection.

Additionally, 11 open-ended questions, including topics such as **sustainable behavior** and **climate change** , are part of this tool. To measure the changes in individual health perceptions of people residing near the areas of intervention of the GGR Cultivating Cities, the Environmental Health Citizen Interview Tool will be used in the areas of intervention in the GGR Cultivating Cities before and after the NbS. The interviews will be audiotaped, to allow for the open-ended questions and answers to be transcribed verbatim. Moreover, data will be pseudo-anonymised (ie, names will not be retrieved). Transcripts will be analyzed carefully and codes will be administered to meaningful sections. Further, a thematic analysis approach will be applied. To ensure methodologic quality strategies to achieve trustworthiness will be established. The latter is guided by the four concepts as described by Lincoln and Guba (1985): dependability, credibility, transferability, and confirmability. To achieve this an audit trail will be kept and researcher triangulation will be applied during data collection and analysis.

**a. KEY ASPECTS WHEN CONDUCTING QUALITATIVE RESEARCH**

1. **inductive process:** although the items in the spider web are inherently deductive, the interviewer must approach the interview in an inductive way. Meaning that the answers of the participant will guide the interview, not the structure of the tool.
2. **importance of context:** during both data collection and analysis the researcher should be aware of contextual factors influencing the behavior and the answers given by participants. The Environmental Health Citizen Interview Tool is to be used in a naturalistic inquiry, as a consequence the researcher should consider her own influence. memos should be made during or right after the interview.
3. **importance of the emic perspective:** data gathered with this tool will be ‘subjective’, hence linked to the ‘subject’. Therefore the interviewer should never assume to know what the participant ‘means’. The interviewer should listen and ask questions as if they were to have no knowledge at all.
4. **importance of reflexivity:** the interviewer should be aware of their own preconceptions and detach from them when conducting the interview.

**b. ENVIRONMENTAL HEALTH CITIZEN INTERVIEW TOOL FIELDWORK ACTIVITY STEP BY STEP**

1. This protocol is written for two people executing the interview: Interviewer one

referred to as I1 speaks the local language; interviewer two referred to as I2 is a

researcher and provides backup and logistic support.

2. Define the target area in which you will approach participants. In addition, when

possible identify diverse populations and approach them beforehand to schedule an

interview if possible.

3. Introduce yourself as part of the GoGreenRoutes project and ask people if they want

to participate in a 15 to 20min interview about how they feel about the green area

they are in, also introduce I2 (I1).

4. If the person agrees to participate, give them a paper version of the information letter,

explain the content if necessary and invite the participant to ask questions.

5. Let the participant sign an informed consent form. This is an ethical requirement,

make sure to obtain written informed consent of each participant.

6. Start the interview by asking participants to score the items, therefore give them the

paper and a pencil to circle the scores and provide assistance when needed (I1).

start audio recording the interview and insert the scores in Excel, using one tab for

each participant and generate a spiderweb (I2).

7. Show the spiderweb to the participant and ask them to explain why they scored as

they did, and provide information on the meaning of the spiderweb (I1).

8. Start the open ended part of the semi-structured interview (I1). Do not rigidly follow

the questions listed in the document, let participants explain and move to questions

that promote the natural flow of the conversation. Ask additional questions to clarify

when needed (see also a. for additional info).

9. Stop the audio recording (I2).

10. Ask the participant to fill in the form with socio-demographic questions and explain

the necessity of this information for the research (Gender, Inclusion and Diversity

aspects of this type of data) (I1).

11. Thank the participant for their participation and say goodbye (I1).

12. Insert the socio-demographic data in the excel file. Use a different row for each

participant in the same socio-demographic tab (I2). Destroy the paper form with

socio-demographic information.

13. Repeat the process until you have interviewed approximately 20 people. Write a

reflective memo including the following aspects (I1 and I2):

- describe your own selection bias (what type of participant is more likely to be approached by you)
- describe your own personal view about the greenspace you are in and how this might impact people’s well-being (this potentially influences your questioning and attitude/appearance).
